# Efficacy of a comprehensive binary classification model using a deep convolutional neural network for wireless capsule endoscopy

**DOI:** 10.1038/s41598-021-96748-z

**Published:** 2021-09-01

**Authors:** Sang Hoon Kim, Youngbae Hwang, Dong Jun Oh, Ji Hyung Nam, Ki Bae Kim, Junseok Park, Hyun Joo Song, Yun Jeong Lim

**Affiliations:** 1 Department of Internal Medicine, Dongguk University Ilsan Hospital, Dongguk University College of Medicine, Dongguk-ro 27 Ilsandong-gu, Goyang, 10326 Republic of Korea; 2 Department of Electronics Engineering, College of Electrical and Computer Engineering, Chungbuk National University, Cheongju, Republic of Korea; 3 Department of Internal Medicine, Chungbuk National University College of Medicine, Cheongju, Republic of Korea; 4 Department of Internal Medicine, Digestive Disease Center, Institute for Digestive Research, Soonchunhyang University College of Medicine, Seoul, Republic of Korea; 5 Department of Internal Medicine, Jeju National University School of Medicine, Jeju, Republic of Korea

**Keywords:** Gastroenterology, Gastrointestinal bleeding, Inflammatory bowel disease, Machine learning

## Abstract

The manual reading of capsule endoscopy (CE) videos in small bowel disease diagnosis is time-intensive. Algorithms introduced to automate this process are premature for real clinical applications, and multi-diagnosis using these methods has not been sufficiently validated. Therefore, we developed a practical binary classification model, which selectively identifies clinically meaningful images including inflamed mucosa, atypical vascularity or bleeding, and tested it with unseen cases. Four hundred thousand CE images were randomly selected from 84 cases in which 240,000 images were used to train the algorithm to categorize images binarily. The remaining images were utilized for validation and internal testing. The algorithm was externally tested with 256,591 unseen images. The diagnostic accuracy of the trained model applied to the validation set was 98.067%. In contrast, the accuracy of the model when applied to a dataset provided by an independent hospital that did not participate during training was 85.470%. The area under the curve (AUC) was 0.922. Our model showed excellent internal test results, and the misreadings were slightly increased when the model was tested in unseen external cases while the classified ‘insignificant’ images contain ambiguous substances. Once this limitation is solved, the proposed CNN-based binary classification will be a promising candidate for developing clinically-ready computer-aided reading methods.

## Introduction

The small intestine is the hardest part of the gastrointestinal (GI) tract to examine using traditional endoscopic and radiologic techniques, owing to their length and tortuous course^1^. In the past few decades, several imaging techniques have been substantially developed to provide images with high temporal and spatial resolutions. Among them, the wireless capsule endoscopy (WCE) is a widely used non-invasive and patient-friendly endoscopic exploration of the entire small bowel with complete video-facilitating detection and monitoring of lesions that was introduced by Iddan et al. in 2000^2^. The European Society of Gastrointestinal Endoscopy (ESGE) recommended WCE as the initial evaluation and diagnostic method for patients with obscure gastrointestinal bleeding (OGIB), suspected Crohn’s disease, and negative ileo-colonoscopy findings^3^.

However, after two decades since its introduction, the way of reading WCE has not changed, which is very time intensive and prone to reader error. Conventionally, it is performed manually by expert gastroenterologists who check the entire recorded video, which is approximately 10 to 13 h long with an average reading time of approximately 30 to 40 min^4,5^. This time-consuming method acquires and generates thousands of images and videos in which relevant findings are only available in few frames^6,7^. Moreover, the fast video playback and extended reading time lead to the loss of the reader’s concentration, which may increase the chance of missing lesions. In addition, the manual reading of WCE images is limited by the insufficient number of gastroenterologists who have adequate experience with small bowel diseases.

Artificial intelligence (AI) has played an increasing role in the advancement of clinical practices. Specifically, the use of AI in capsule endoscopy has gained attention for its potential to automatically detect diseases from the video and shorten WCE reading time^8^. Previous studies have reported the application of the convolutional neural network (CNN), which is the main deep learning algorithm for image analysis, in reading WCE images. Some CNN-based algorithms have been successful for detecting a variety of small bowel diseases, including ulcers, polyps, Crohn’s disease, angioectasia, and bleeding^9–17^. However, the utilization of CNN-based reading algorithms remains on the research stage despite their advancements, which includes their potential detection of various small bowel lesions with high accuracy in unseen images^18,19^. At present, the complete application of this technology has not yet been fully realized despite various efforts such as increasing the number of images for AI learning.

Therefore, we aim to create a new CNN-based model that is practical and clinically ready-to-use. As part of that effort, we developed a CNN-based binary classification algorithm that could categorize images into clinically meaningful lesions and those that are not. With this, the reading time can be drastically reduced since only AI-selected images need to be reviewed by gastroenterologists. Hence, we developed a CNN-based binary classification model for capsule endoscopy and validated it with external unseen cases.

## Methods

### Preparation of images for AI training, validation, and internal testing

The study protocol was approved by Dongguk University Ilsan Hospital Institutional Review Board (No. 2018-00-009-002). Eighty-four capsule endoscopy (CE) videos that were taken from June to December 2019 were retrospectively acquired from a single institute (Dongguk University Ilsan Hospital) employing a Mirocam MC1600 device (Intromedic, Seoul, South Korea) to be used for training and development of the CNN-based AI. These images were extracted in PNG format (320 $\times$ 320 pixel) using MiroView 4.0. After data anonymization, all small bowel images were reviewed and labeled manually into 7 subgroups (normal mucosa, bile predominant, air bubbles, debris, inflamed mucosa, atypical vascularity, and bleeding) by three experienced gastroenterologists. Two expert gastroenterologists (clinical professors with more than 3 years of experience in CE, SHK, and DJO) from Dongguk University Ilsan Hospital independently read 84 CE videos image-by-image. Then, these were reviewed by a senior gastroenterologist (with more than 10 years of experience in CE, YJL) to see if there were inconsistent readings. Then, these images were binarily categorized as ‘Significant’ if it contains a lesion of clinical significance, such as inflamed mucosa, atypical vascularity, or bleeding, and ‘Insignificant’ if the image does not present any abnormalities, including normal mucosa, bile, air bubbles, or debris. A total of 400,000 images, consisting of 200,000 clinically significant images and 200,000 insignificant images, were randomly selected for AI training, validation, and internal testing. In this study, although CE images were collected retrospectively, we obtained informed consent from all patients participating in the study. Moreover, we obtained the informed consent for minor participants (age 18 or younger) from their legally authorized representatives. All research protocols were conducted following relevant guidelines and regulations.

### Distribution of collected CE images

**Figure 1:**
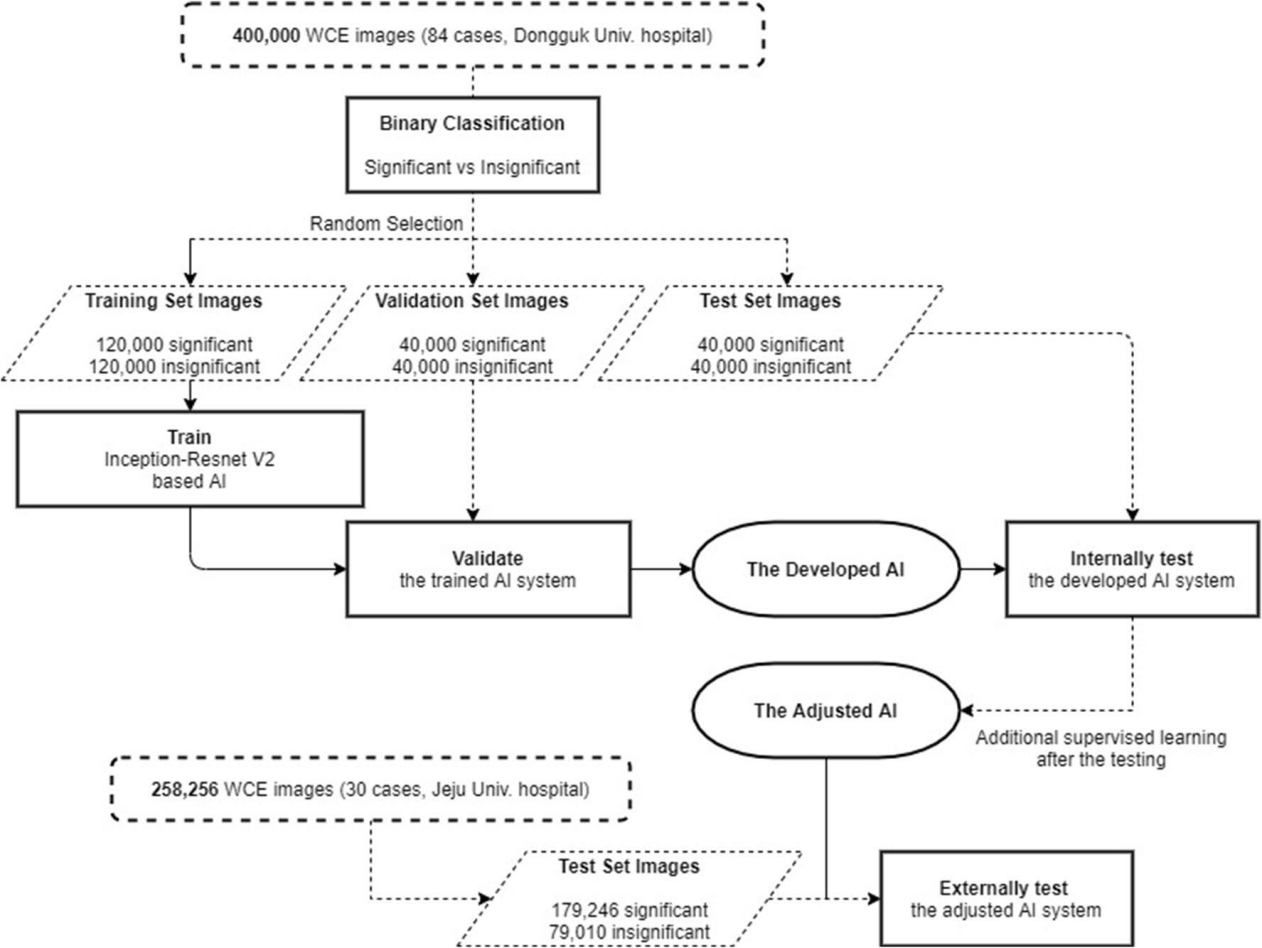
Flowchart of the study design.

The labeled images (n = 400,000) were then classified into the following three categories (Fig. 1): training (n = 240,000, 60%), validation (n = 80,000, 20%), and internal testing (n = 80,000, 20%). It was necessary to set the best parameters for 80,000 (20%) images used for validation to ensure optimal diagnostic accuracy. Moreover, these images for validation were also used for additional training. Therefore, a total of 320,000 images were used in the development of the algorithm, while the remaining 80,000 (20%) images were used for internal testing as they were not used for training.

### Collection of external hospital images for testing of the developed AI model

We retrospectively collected 30 CE videos from another medical hospital (Jeju National University Hospital) with the approval of its Institutional Review Board (Jeju National University Institutional Review Board, ‘JEJUNUH 2019-11-010’). A total of 258,256 images (significant: 179,246; insignificant: 79,010) were extracted from videos using Mirocam MC1600 and the same image extraction protocol. In addition, the three expert gastroenterologists who participated in the image classification for AI training were also the ones who classified the collected images into two groups (‘significant’ and ‘insignificant’) based on the same criteria used for training. With this, it was possible to evaluate the diagnostic accuracy of the AI model for ‘unseen’ data acquired from a non-trained hospital.

### Development of the CNN-based AI for auxiliary reading model

**Figure 2:**
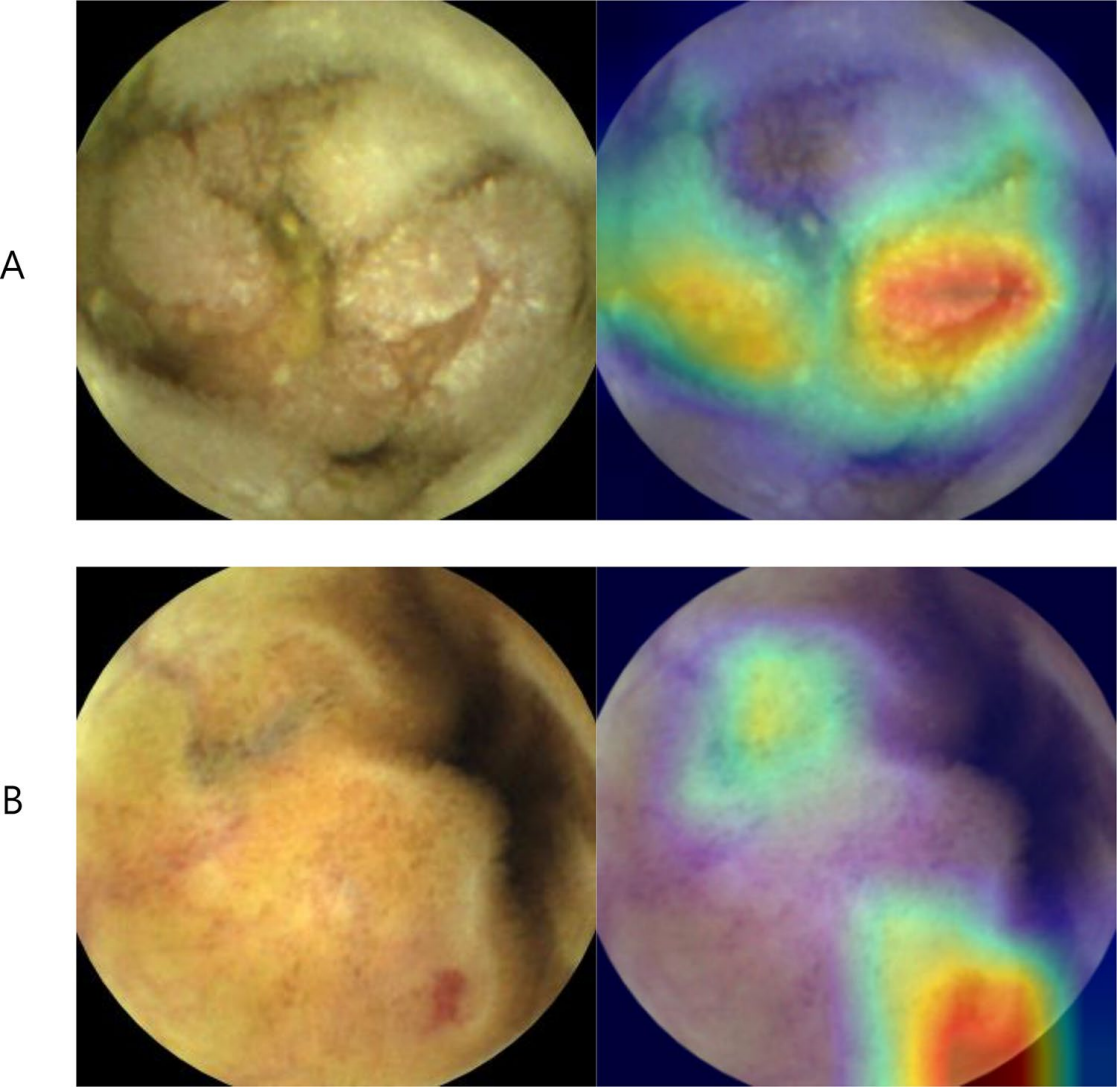
Class activation map of significant lesions. (Capsule endoscopy image taken by Mirocam MC1600, processed by MiroView 4.0—http://www.intromedic.com/eng/main).

We employed the Inception-Resnet-V2 model in the TensorFlow-Slim (TF-Slim) library, which is known for its light and efficient multi-level feature extraction of an inception module and deeper layers of a Resnet module to train an AI, for the binary classification of CE images. For better recognition accuracy, we applied transfer learning using the pre-trained model from imageNet dataset. First, we used the learning rate of 0.01 for training the last ‘logits’ layer during 10,000 steps. Then, we trained all the layers with the learning rate of 0.0001 during 200,000 steps. Similarly with original InceptionResnetV2 model, we did not use dropout, and we used the rectified linear unit (ReLU) as a transfer function. The elapsed time to transform the image data into TFrecord (binary data rapidly readable in Tensorflow) for 80,000 test images was 2621.208 s. Meanwhile, the processing time for evaluating the transformed data was 431.258 s. Therefore, the proposed AI showed a processing speed of 26.208 fps. Class activation maps (CAP) were drawn based on a channel-wise aggregation method (Fig. 2), which employs a global average pooling (GAP) for each channel after the final convolutional layer stage that demonstrates pixel-wise predicted values for the class, to analyze which regions influenced the selection of the final class. We were able to verify that predictions made by the trained deep learning network were similar to those of endoscopists using CAP in which regions with clinical significance were indicated by the red color. From Fig. 2, the jet color map shows normalized prediction in which the reddish and bluish colors refer to 1 and 0, respectively. This figure describes the approximate mechanism of the developed AI.

### Outcomes and statistical analysis

The crucial results, including the area under the receiver operating characteristic (ROC) curve, sensitivity, specificity, and accuracy of the CNN-based AI model, indicate whether each image possessed lesions of clinical significance. The trained model shaped the region of clinical significance and described the probability score of the lesion (range: 0–1). We verified the score threshold for the best output through the validation process. Data were analyzed using the Statistical Package for Social Science, Ver 20.0 (SPSS Inc., Chicago, IL, USA).

## Results

### Study population

**Figure 3:**
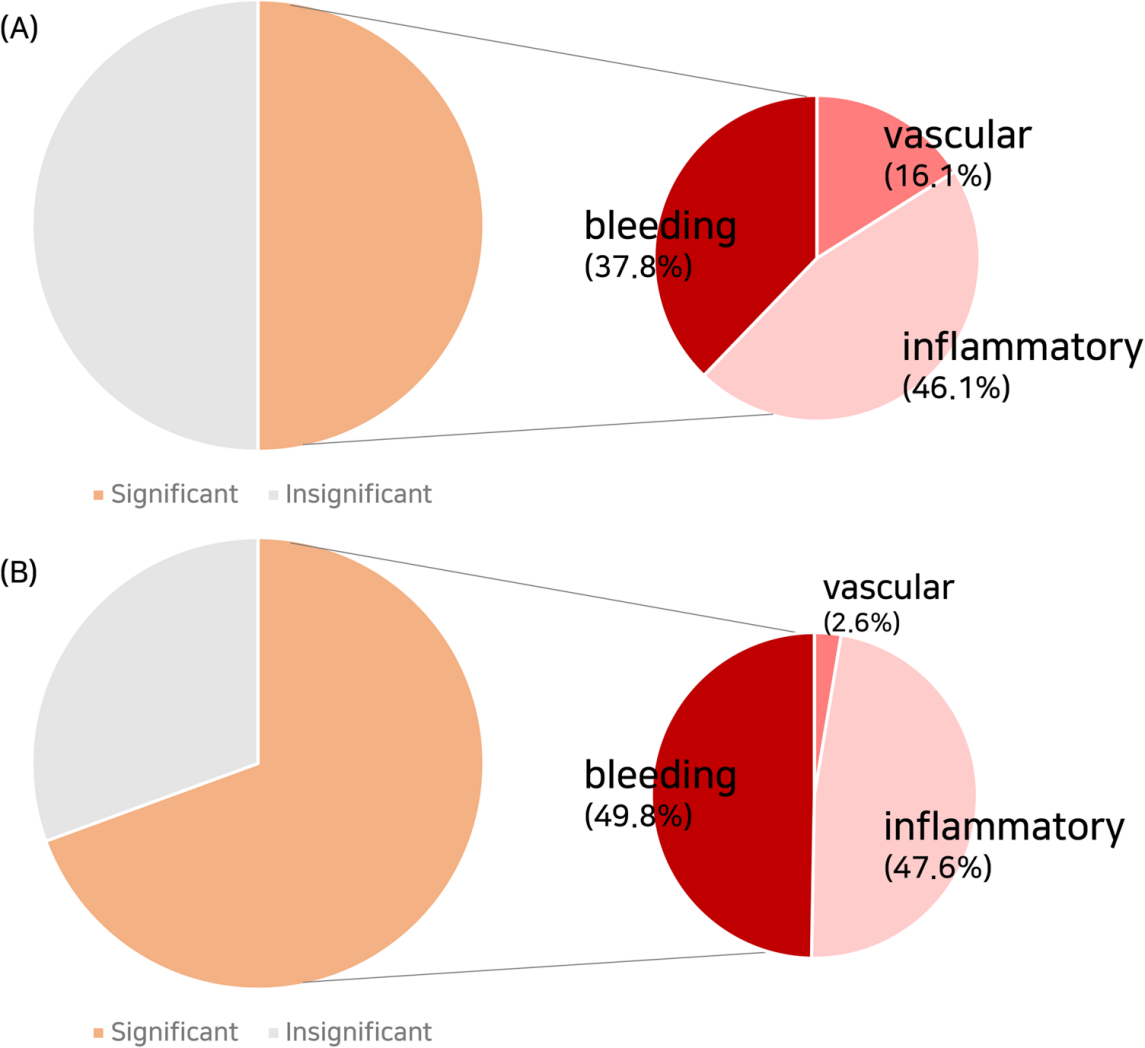
Composition of two datasets, such as the (**A**) learning and internal test and (**B**) external test.

The clinical and demographic characteristics of images used for algorithm training and external testing are shown in Table 1. Meanwhile, the composition of the two datasets is described in pie charts (Fig. 3). The average age of the cases used for training is 49.84 years (range: 16–92 years), while 52.31 years for the external testing (range: 16–76 years). In addition, the indications for CE for both datasets were obscure GI bleeding, inflammatory bowel disease, small bowel tumors, and others. Moreover, significant and insignificant images were randomly selected for both dataset (200,000 each for the training dataset and 179,246 and 79,010 for the external test dataset). Based on the table below, the relative proportions of inflammatory lesions and air bubbles were high for the training dataset. Moreover, the significant images with inflammatory lesions or bleeding were included relatively more than others. Therefore, there was a difference in image composition between data used for training and data used for external testing.

**Table 1:** Clinical and demographic characteristics of cases.

Characteristics	Training dataset (n = 84)	External test dataset (n = 30)
No. of images	400,000	256,591
Age (years), mean (± SD)	49.84 (± 19.17)	52.31 (± 19.08)
Sex, male	55 (65.5%)	18 (60.0%)
**Indication of capsule endoscopy**		
Obscure GI bleeding	43 (51.2%)	19 (63.3%)
Small bowel tumors	10 (11.9%)	3 (10.0%)
Inflammatory bowel disease	30 (35.7%)	8 (26.7%)
Others	1 (1.2%)	0 (0.0%)
**Types of included images**		
Normal mucosa	42,729	21,317
Bile	58,812	18,196
Air bubbles	87,494	6,550
Debris	10,965	32,947
Vascular	32,116	4,733
Inflammatory	92,297	85,387
Bleeding	75,587	89,126
**Binary classification of included images**		
Significant	200,000	179,246
Insignificant	200,000	79,010

### Internal test result: binary classification capability through optimal threshold setting

The calculated probabilities of significance and results of class map activation for all images were reviewed by three gastroenterologists who contributed to the labeling of training images (Table 2). The difference in the classification ability for each cut-off probability score was reviewed by endoscopists who agreed to set the cut-off threshold at 0.5 after examining the concordance between manual classification results and calculated probability values for validation images. Based on this threshold, the AI system's sensitivity was 98.691%, specificity was 97.208%, and accuracy was 97.946% for the selected internal test set of images. The area under the curve (AUC) value in the internal testing was 0.998.

**Table 2:** Changes in the classification ability for each cut-off probability score tested on the validation set of images.

Cut-off value (probability score)	Sensitivity (%)	Specificity (%)	Accuracy (%)
0.1	0.893	0.999	0.946
0.2	0.928	0.999	0.963
0.3	0.949	0.997	0.973
0.4	0.963	0.994	0.978
0.5	0.973	0.988	0.980
0.541*	0.977	0.985	0.981
0.6	0.982	0.978	0.980
0.7	0.989	0.957	0.973
0.8	0.994	0.911	0.953
0.9	0.998	0.784	0.891

(*) is the estimated value according to the Youden index.

### External test result: accuracy drop for new and unseen cases

**Figure 4:**
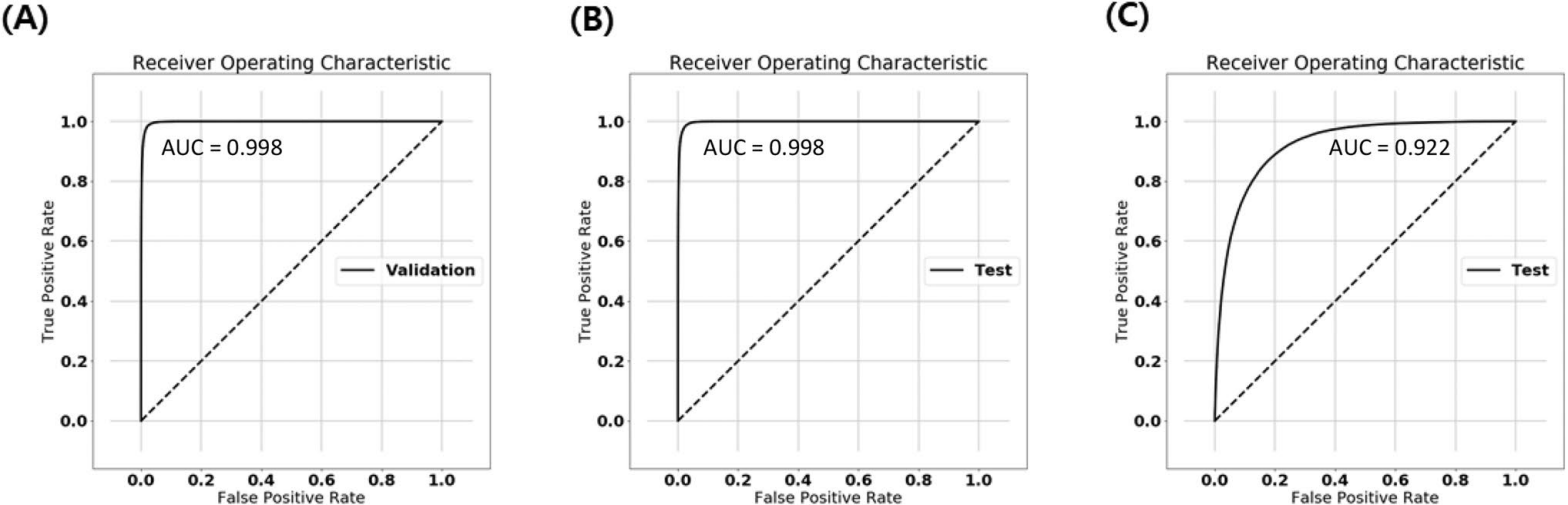
Receiver operating characteristic curves of (**A**) internal validation, (**B**) internal testing, and (**C**) external testing.

The accuracy of the developed algorithm that was tested with an external set of images was reduced to 85.470%, with a sensitivity of 89.684% and a specificity of 75.994%. In addition, the AUC value was 0.922 in the external testing. Moreover, the ROC for the external testing compared to the internal validation process is demonstrated in Fig. 4.

The performance of the CNN model at each validation level is described in Table 3. The sensitivities of the model at the preset cut-off value of 0.5 for significant lesions were 97.3% for the internal validation, 97.2% for the internal testing, and 89.7% for the external testing.

**Table 3:** Performance of binary classification model at each validation level.

	Internal validation	Internal testing	External testing
No. of images	80,000	80,000	256,591
AUC	0.99794	0.99775	0.92194
Accuracy, %	98.067	97.946	85.470
Sensitivity, %	97.322	97.208	89.684
Specificity, %	98.817	98.691	75.994

All performance outcomes were calculated when a cut-off value of 0.5 was applied.

### Additional, comprehensive sensitivity analysis

**Figure 5:**
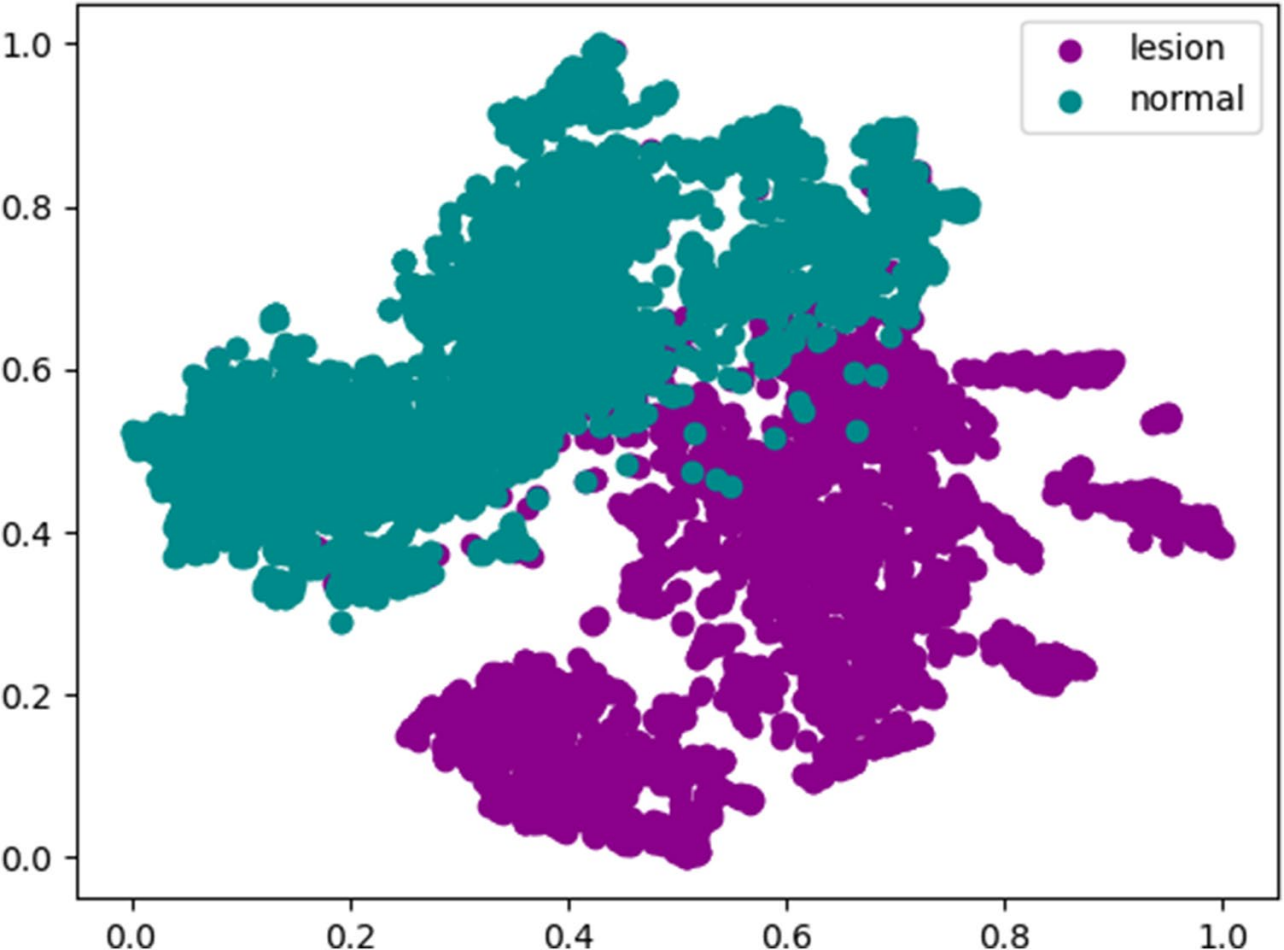
t-SNE plot demonstrating cluster assignment of the CNN model.

By randomly selecting 8,000 images from 400,000 images, we performed t-SNE (t-Stochastic Neighbor Embedding) for additional sensitivity analysis. As shown in Fig. 5, images are separated into two groups quite well in the deep learning feature.

### Difference in classification performance according to image type

We compared the ability of the CNN model in classifying images into significant and insignificant groups compared to the manual classification done by endoscopists (Table 4). A total of 160,849 images out of the 179,246 ‘significant’ images classified by endoscopists were classified correctly by the CNN, leading to a false-negative rate of 10.3%. On the other hand, 60,770 images were correctly classified as insignificant from the total of 79,010 ‘insignificant’ images classified by endoscopists, showing a false-positive rate of 23.1%. We found that achieving specificity was more challenging than sensitivity in the binary classification process by the CNN model.

**Table 4:** Classification results of manually labeled images by the CNN.

CNN classification (external testing)	Classification by endoscopists		Total	
	Significant	Insignificant		
Significant	160,849	18,240	179,089	**PPV 89.8%**
Insignificant	18,397	60,770	79,167	**NPV 76.8%**
Total	179,246	79,010		
	**Sensitivity 89.7%**	**Specificity 76.9%**		

The contents marked in bold indicate the accuracy of the algorithm.

*PPV* positive predictive value, *NPV* negative predictive value.

**Figure 6:**
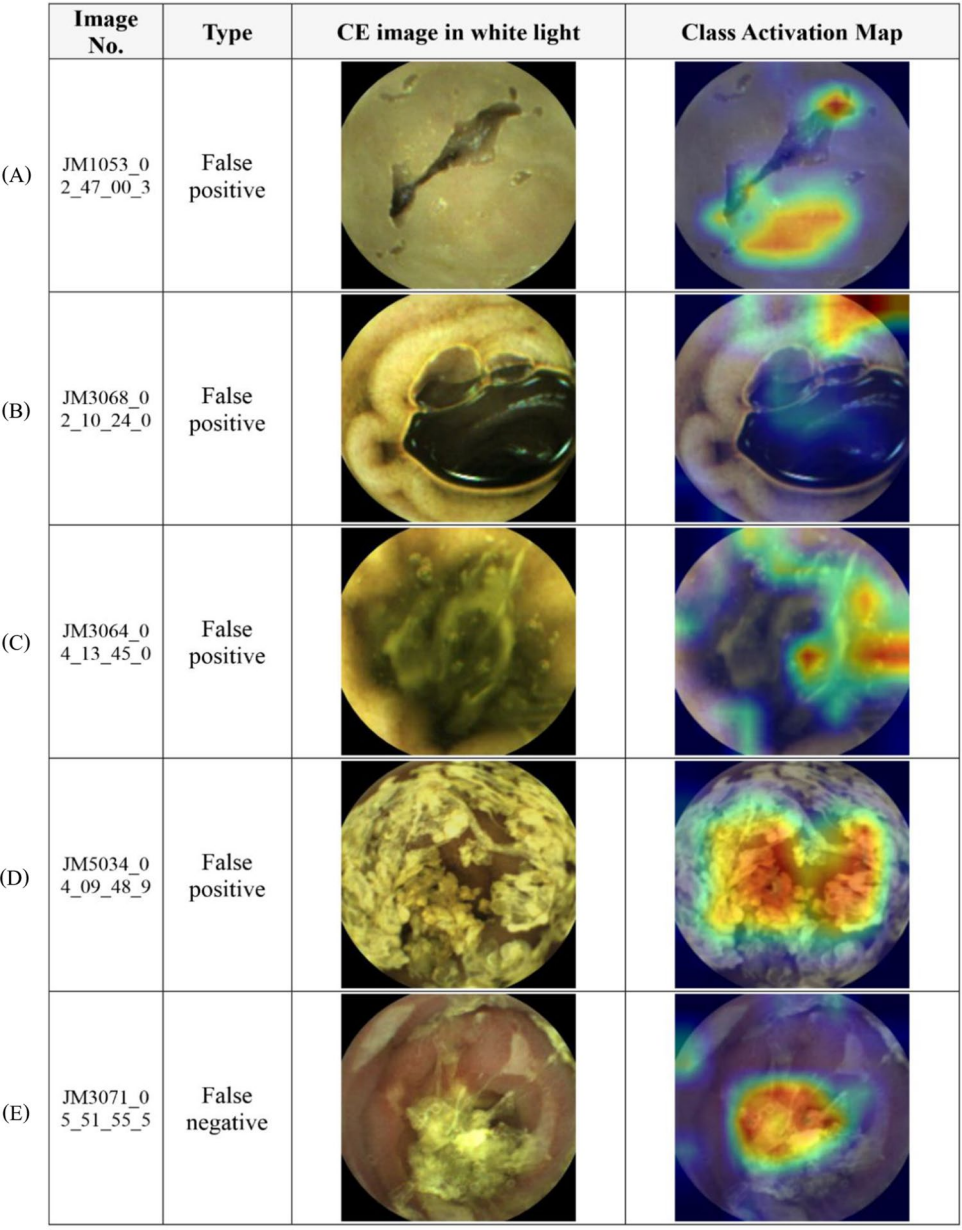
Examples of Several Class Activation Maps of Misclassified Images. (**A**) Is a case of incorrect detection of induration caused by capsule and small bowel mucosa adhesion. (**B**) Is misclassified as 'significant' for bends around a large bubble. (**C**) Is a case that was successful in discriminating bile and debris but misclassified as 'significant' for a peripheral dimmed lesion with a flow of bile. (**D**) Is misclassified semi-solid debris as 'significant' lesion. (**E**) Is a case showing inappropriate higher activation in debris rather than focusing on inflammation and hyperemia of the mucosa. (Capsule endoscopy image taken by Mirocam MC1600, processed by MiroView 4.0—http://www.intromedic.com/eng/main).

Among several subtypes of insignificant images, the subtype that was more prone to misreading was then analyzed. As a result, we found that images were more likely to be classified as significant even without abnormalities in 40.3% of cases when air bubbles occupied most of the image (Table 5). For example, as shown in Fig. 6, although only air bubbles and normal mucosa were present in the small bowel lumen, the CNN class activation map pointed out that the area without significance possessed a lesion with a probability score above the threshold. On the other hand, for significant images, while the probability of misreading was lower, images with bleeding were more error-prone than other etiologies (Table 6).

**Table 5:** False positive rate by the CNN model according to subtypes of insignificant images.

	Subtypes of insignificant images				
	Mucosa	Bile	Air bubbles	Debris	Total
Total No	21,317	18,196	6550	32,947	79,010
False (+) No	5051	3172	2637	7380	18,240
False (+) %	23.69%	17.43%	40.26%	22.40%	23.09%

**Table 6:** False negative rate by the CNN model according to subtypes of significant images.

	Subtypes of significant images			
	Inflamed	Vascular	Bleeding	Total
Total No	85,387	4733	89,126	179,246
False (−) No	2220	351	15,826	18,397
False (−) %	2.60%	7.42%	17.76%	10.26%

## Discussion

In this study, we developed a practical CNN-based AI model that performs comprehensive binary classification. The accuracy of the model was tested using acquired CE images from two institutions, including where the AI learning was conducted (Dongguk University Ilsan Hospital) and an independent external hospital (Jeju National University Hospital). To the best of our knowledge, this is the first AI model developed and tested using Mirocam capsule image-set. The diagnostic accuracy during validation and internal testing was 98%, with an excellent AUC of 0.99. However, the accuracy of the model decreased by 10% when tested on the external hospital data with an AUC drop of 0.07.

It is essential to detect various gastrointestinal pathologies for the automated AI reading in CE. Moreover, a broad spectrum of diseases can be present in the small intestine, making it difficult for an AI algorithm to classify them correctly in a multi-class manner. In addition, it is difficult to develop a successful model using AI learning because of several problems, including data imbalance between normal and lesion images. In addition, when two or more lesions are mixed in a single image without individually annotating them may lead to incorrect learning and inappropriate reading. In this regard, a CNN-based model that can perform binary classification is considered to be a practical and achievable concept. The sensitivity and positive predictive value (PPV) are important parameters when evaluating such a model because it was used in selecting images with relevant findings that were being provided to endoscopists. Our algorithm showed a high sensitivity of 89.7% and a PPV of 89.8% even for unseen external cases, indicating lesser reading time and reduced rate of misreadings.

Furthermore, the AI-assisted image recognition and classification technology is auspicious because reading CE videos is a time intensive and tedious process. In the last 2 years, previous studies on the automated reading of CE have been published, including a study showing very high sensitivity and specificity by detecting angioectasia using a CNN model proposed by Leenhardt et al.^14^, a model published by Aoki et al. that judged erosions and ulcerations with an accuracy of 90%^12^, and a study by Iakovidis et al. using weakly supervised learning AI model for diagnosing a variety of pathologies^20^. In addition, Ding et al.^17^ collected over 100 million CE images from 77 hospitals and trained AI with 158,235 images from 1970 cases and evaluated whether an accurate diagnosis of various types of lesions was possible.

Nevertheless, most AI models for CE are considered insufficient for clinical use because they only detect one or two specific disease entities with insufficient diagnostic accuracy despite training these models with a relatively large number of images. Indeed, a study design that was tested using images from the same institution where the training images were gathered (even though it was multi-center based) raised questions whether the developed AI model will have high accuracy when tested with images from external hospitals. In this study, we tested the algorithm in a ‘patient isolation’ manner with unseen images from an external hospital to test its real-world efficacy. We found that there was still a difference in AUCs between the internal and external tests.

WCE was taken as a motion video in which 50,000 images were saved per session. Each CE image may contain either pure significant (or insignificant) content or significant lesions mixed with some insignificant materials. Most AI models are specialized in detecting ‘lesions’ only and get confused when the lesion is widely covered by insignificant content. In this regard, our model also showed that insignificant images were more likely to be classified as false positives especially images with poor bowel preparation (which contains bile, bubbles, and debris). The AI model should also be trained to recognize ‘insignificant’ contents correctly and show an accurate probability value even with images of mixed significance, indicating that an improved algorithm that can separate groups of consecutive low-quality images and exclude these groups from AI reading may enhance the reading accuracy and reduce the presentation of unnecessary images to ‘human’ interpreter. Therefore, promoting the ability to judge the quality of bowel preparation by the CNN-based model is important.

The results showed that the diagnostic accuracy of AI tested with unseen external data decreased by approximately 10%, owing to the demographic difference of patients undergoing CE at each hospital. The trained AI model might have also faced completely new endoscopic images that were not experienced during training. For the AI detection model for CE to be used in real-world clinical practice, the algorithm's sensitivity must be at least 99%. In this study, although the internal test result was very encouraging (sensitivity of 97.3%), the relatively lower sensitivity (89.7%) was observed for external unseen cases that was below the cut-off value for commercialization.

Based on the study conducted by Segui et al., increasing training images by ten times improved the diagnostic accuracy by 3% in the WCE analysis^21^. Moreover, the following accuracy test with CE images of two hospitals is expected to improve over 95% by adding supervised learning using the unseen external data. However, there are still concern regarding ‘catastrophic forgetting’ in which the accuracy of the diagnosis for the dataset of the initial training from a single institution may be reduced when the AI is additionally trained with a large number of images from other institutions. In addition, manually annotating massive data for CE images is also a laborious, error-prone, and expensive process that may lead to huge label bias. Therefore, a large number of multi-center based training images will not necessarily lead to a clinically-ready AI model.

We believe that it is necessary to form an optimized training image set that is suitable for the health environment of a regional community where capsule endoscopy is performed to advance to a stage of multi-class reading. In this study, we considered the data balance when setting up the learning image database since we knew that the significant images were too small even in hundreds of thousands of capsule images. Therefore, we set the ratio of pathological and nonpathological images to 1:1 for AI training to detect pathological images sensitively and deliver them to a human reader without any loss. However, by properly adjusting ‘Significant’ and ‘Insignificant’ ratios of images as well as the compositions of lesions (for example, the ratio among inflammatory, vascular, bleeding, and small bowel tumors), we expected a more rational AI reading outcome. Regarding the optimal image composition ratio for each regional community, additional studies are still needed.

This study has several limitations. First, the AI training was based on images from a single institution. Second, the labeling of images was done by three gastroenterologists, which may include some inter-observer differences. In addition, it only used retrospectively collected data without utilizing clinical data during learning and feedback processes.

Securing the reliability of AI decision-making is very important to whether it can be used in the real world. There are questions about which AI systems should be trusted more or less, and performing a comprehensive uncertainty quantification and trustworthiness is a very difficult process^22^. Accuracy and AUC may be representative metrics, but they are still not perfect. Developing a format that can quantify the uncertainty of artificial intelligence that considers both the dataset and the trustworthiness of the inner algorithmic workings would be one of many future challenges.

In summary, we demonstrated the practical applicability of a CNN-based comprehensive binary classification model in the small bowel CE, which is a promising tool that can be used in everyday practice in the near future.

## Supplementary Information

Supplementary Table 1.

## Data Availability

The datasets analyzed in the current study are available upon reasonable request from the corresponding authors.

## Abbreviations

WCE: Wireless capsule endoscopy
CNN(s): Convolutional neural network(s)
TF-Slim: TensorFlow-Slim
AUC: Area under the curve
ESGE: European Society of Gastrointestinal Endoscopy
GI: Gastrointestinal
AI: Artificial intelligence
CE: Capsule endoscopy
CAP: Class activation map
GAP: Global average pooling
ROC: Receiver operating characteristic
ReLU: Rectified linear unit
t-SNE: t-stochastic neighbor embedding

## References

[1] Aktas H, Mensink PB (2012). Small bowel diagnostics: Current place of small bowel endoscopy. Best Pract. Res. Clin. Gastroenterol..

[2] Iddan G, Meron G, Glukhovsky A, Swain P (2000). Wireless capsule endoscopy. Nature.

[3] Pennazio M (2015). Small-bowel capsule endoscopy and device-assisted enteroscopy for diagnosis and treatment of small-bowel disorders: European Society of Gastrointestinal Endoscopy (ESGE) clinical guideline. Endoscopy.

[4] Mishkin DS (2006). ASGE technology status evaluation report: Wireless capsule endoscopy. Gastrointest. Endosc..

[5] Koulaouzidis A, Iakovidis DK, Karargyris A, Plevris JN (2015). Optimizing lesion detection in small-bowel capsule endoscopy: From present problems to future solutions. Expert Rev. Gastroenterol. Hepatol..

[6] Lee NM, Eisen GM (2010). 10 years of capsule endoscopy: An update. Expert Rev. Gastroenterol. Hepatol..

[7] Rondonotti E (2012). Can we improve the detection rate and interobserver agreement in capsule endoscopy?. Dig. Liver Dis.: Off. J. Ital. Soc. Gastroenterol. Ital. Assoc. Stud. Liver.

[8] Hricak H (2018). 2016 New horizons lecture: Beyond imaging-radiology of tomorrow. Radiology.

[9] Xiao, J. & Meng, M. Q. A deep convolutional neural network for bleeding detection in wireless capsule endoscopy images. In *Conference Proceedings: Annual International Conference of the IEEE Engineering in Medicine and Biology Society. IEEE Engineering in Medicine and Biology Society. Annual Conference* 639–642. 10.1109/embc.2016.7590783 (2016). 10.1109/EMBC.2016.7590783 28268409

[10] Fan S, Xu L, Fan Y, Wei K, Li L (2018). Computer-aided detection of small intestinal ulcer and erosion in wireless capsule endoscopy images. Phys. Med. Biol..

[11] Alaskar H, Hussain A, Al-Aseem N, Liatsis P, Al-Jumeily D (2019). Application of convolutional neural networks for automated ulcer detection in wireless capsule endoscopy images. Sens. (Basel, Switz.).

[12] Aoki T (2019). Automatic detection of erosions and ulcerations in wireless capsule endoscopy images based on a deep convolutional neural network. Gastrointest. Endosc..

[13] Klang E (2020). Deep learning algorithms for automated detection of Crohn's disease ulcers by video capsule endoscopy. Gastrointest. Endosc..

[14] Leenhardt R (2019). A neural network algorithm for detection of GI angiectasia during small-bowel capsule endoscopy. Gastrointest. Endosc..

[15] Aoki T (2019). Automatic detection of blood content in capsule endoscopy images based on a deep convolutional neural network. J. Gastroenterol. Hepatol..

[16] Tsuboi A (2020). Artificial intelligence using a convolutional neural network for automatic detection of small-bowel angioectasia in capsule endoscopy images. Dig. Endosc.: Off. J. Jpn. Gastroenterol. Endosc. Soc..

[17] Ding Z (2019). Gastroenterologist-level identification of small-bowel diseases and normal variants by capsule endoscopy using a deep-learning model. Gastroenterology.

[18] Hwang Y, Park J, Lim YJ, Chun HJ (2018). Application of artificial intelligence in capsule endoscopy: Where are we now?. Clin. Endosc..

[19] Park J (2019). Recent development of computer vision technology to improve capsule endoscopy. Clin. Endosc..

[20] Iakovidis DK, Georgakopoulos SV, Vasilakakis M, Koulaouzidis A, Plagianakos VP (2018). Detecting and locating gastrointestinal anomalies using deep learning and iterative cluster unification. IEEE Trans. Med. Imag..

[21] Seguí S (2016). Generic feature learning for wireless capsule endoscopy analysis. Comput. Biol. Med..

[22] Cheng M, Nazarian S, Bogdan P (2020). There is hope after all: Quantifying opinion and trustworthiness in neural networks. Front. Artif. Intell..

