# Supplementary material for: Efficacy of a comprehensive binary classification model using a deep convolutional neural network for wireless capsule endoscopy
Source: Sci Rep. 2021 Sep 1;11:17479. doi: 10.1038/s41598-021-96748-z (PMC8410868; doi:10.1038/s41598-021-96748-z)
Supplement: Supplementary file 1 — Supplementary Table 1. [file 41598_2021_96748_MOESM1_ESM.docx]

**Supplementary data**

**[Table] Hyperparameters used in training the algorithm.**

| **Initial training of only the last layer from the pre-trained model (ImageNet)** | |
| --- | --- |
| Number of steps | 10,000 |
| Batch size | 24 |
| Learning rate | 0.01 |
| Learning rate decay type | Fixed |
| Optimizer | RMSprop |
| Weight decay | 0.00004 |
| Loss function | Binary Cross-Entropy |
| **Full training of all the layers from the initial training** | |
| Number of steps | 200,000 |
| Batch size | 24 |
| Learning rate | 0.0001 |
| Learning rate decay type | Fixed |
| Optimizer | RMSprop |
| Weight decay | 0.0004 |
| Binary Cross-Entropy | Binary Cross-Entropy |
